# Persistent Haematuria Is Associated With Reduced Kidney Survival in Primary Podocytopathies

**DOI:** 10.1111/nep.70252

**Published:** 2026-08-02

**Authors:** Gabriel Ștefan, Nicoleta Petre, Adrian Zugravu, Simona Stancu

**Affiliations:** ^1^ Department of Nephrology University of Medicine and Pharmacy ‘Carol Davila’ Bucharest Romania; ^2^ Department of Nephrology ‘Dr. Carol Davila’ Teaching Hospital of Nephrology Bucharest Romania; ^3^ Department of Pathology University of Medicine and Pharmacy ‘Carol Davila’ Bucharest Romania; ^4^ Department of Pathology ‘Dr. Carol Davila’ Teaching Hospital of Nephrology Bucharest Romania

**Keywords:** focal segmental glomerulosclerosis, haematuria, kidney failure, membranous nephropathy, minimal change disease, podocytopathy

## Abstract

**Aim:**

Haematuria is frequently present in podocytopathies, but its significance and prognostic value are not well described. This study aimed to determine the prevalence and association between persistent haematuria and kidney survival in patients with membranous nephropathy (MN), minimal change disease (MCD) and focal segmental glomerulosclerosis (FSGS).

**Methods:**

We conducted a retrospective cohort study of 236 adults with biopsy‐proven primary podocytopathies. Persistent haematuria was defined as ≥ 5 red blood cells per high‐power field in ≥ 3 consecutive urine specimens within 3 months post‐biopsy. The primary outcome was end‐stage kidney disease (ESKD) requiring chronic renal replacement therapy. Kaplan–Meier analysis and Cox proportional hazards regression were performed.

**Results:**

Among 236 participants (114 MN, 76 MCD, 46 FSGS), 73 (31%) had persistent haematuria. Participants with haematuria had higher proteinuria (5.3 vs. 3.2 g/g, *p* = 0.01) and were more likely to have hypertension (55% vs. 36%, *p* = 0.007). During a median follow‐up of 96 months, 38 patients (16%) reached ESKD. ESKD incidence was significantly higher in patients with persistent haematuria (32% vs. 9%, *p* < 0.001). After adjusting for diagnosis, eGFR, proteinuria, Charlson comorbidity score and histological chronicity score, persistent haematuria remained independently associated with ESKD (hazard ratio: 3.55; 95% CI: 1.78–7.05; *p* < 0.001).

**Conclusion:**

Persistent haematuria is prevalent among patients with podocytopathies and is significantly and independently associated with ESKD. Urinalysis, including microscopic haematuria assessment, is widely available and inexpensive, providing a valuable data point to help formulate patient prognosis.

## Summary at a Glance

In 236 adults with primary podocytopathies (membranous nephropathy, minimal change disease, focal segmental glomerulosclerosis), persistent microscopic haematuria was present in 31% and independently associated with a 3.5‐fold increased risk of end‐stage kidney disease. Haematuria assessment may improve risk stratification in these patients.

## 1 Introduction

Haematuria is associated with incident chronic kidney disease (CKD) in the general population as well as progression of CKD and kidney failure in patients with preexisting CKD from mixed aetiologies [1, 2, 3, 4]. This association, however, is not clearly defined in patients with proteinuric kidney disease or podocytopathies [5, 6, 7, 8]. It has been proposed that haematuria leads to cellular injury due to free heme toxicity or glomerular basement membrane abnormalities [9, 10].

Podocytopathies encompass a heterogeneous group of disorders classified on the basis of histopathologic differences in kidney biopsies and often present with nephrotic syndrome. Even within disease categories, such as minimal change disease (MCD), focal segmental glomerulosclerosis (FSGS) and membranous nephropathy (MN), the disease course is variable and prognostic biomarkers associated with important kidney outcomes are needed. Haematuria is relatively common in previously reported cohorts of patients with podocytopathies (21%–48%) [11, 12, 13, 14]. It is widely measured in the clinic and easy to assess qualitatively by urine dipstick.

In immunoglobulin A nephropathy (IgAN), microscopic haematuria has emerged as an important prognostic biomarker. Sevillano et al. demonstrated that time‐averaged haematuria independently predicted kidney failure, with remission of haematuria associated with stabilisation of kidney function decline [15]. We hypothesised that persistent haematuria, defined by multiple consecutive positive specimens, would be associated with kidney survival in primary podocytopathies. The aims of this study were to (1) determine the prevalence of persistent haematuria in adults with biopsy‐proven podocytopathy and (2) assess the association between persistent haematuria and end‐stage kidney disease (ESKD).

## 2 Materials and Methods

### 2.1 Study Population and Design

We conducted a retrospective cohort study at a tertiary nephrology referral centre. All consecutive adult patients (≥ 18 years) with biopsy‐proven primary MN, MCD or FSGS diagnosed between January 2010 and December 2015 were included. Exclusion criteria included secondary forms of these diseases (lupus nephritis, malignancy‐associated MN, HIV‐associated nephropathy, obesity‐related FSGS, medication‐induced), incomplete baseline data and follow‐up < 12 months. All diagnoses were confirmed by centralised histopathological review. The study was conducted in accordance with the Declaration of Helsinki and approved by the institutional ethics committee (Dr Carol Davila Teaching Hospital of Nephrology No 34/2025).

### 2.2 Clinical and Laboratory Assessment

Baseline clinical data collected at kidney biopsy included age, sex, Charlson comorbidity index and blood pressure. Laboratory parameters included serum creatinine, estimated glomerular filtration rate (eGFR) calculated using the CKD‐EPI equation [16], haemoglobin, serum albumin, C‐reactive protein, cholesterol, triglycerides and proteinuria expressed as protein‐to‐creatinine ratio (g/g). Hypertension was defined as blood pressure ≥ 140/90 mmHg or use of antihypertensive medications. Use of angiotensin‐converting enzyme inhibitors or angiotensin receptor blockers (ACEI/ARB) and immunosuppressive medications was recorded.

### 2.3 Haematuria Assessment

Fresh urine specimens were examined by standard light microscopy within 2 h of collection. Urine samples were centrifuged at 400 g for 5 min according to standard laboratory protocol, and red blood cells were quantified by light microscopy at 400× magnification, averaging counts across multiple high‐power fields (HPF). All analyses were performed in the same institutional laboratory, minimising inter‐observer variability. Red blood cells were quantified and erythrocyte morphology was assessed manually by trained laboratory personnel under light microscopy; automated particle counting was not used. Dysmorphic erythrocytes were systematically recorded as an indicator of glomerular‐origin bleeding and were the predominant morphology among patients with haematuria. Microscopic haematuria was defined as ≥ 5 RBC/HPF, consistent with the threshold used in glomerulonephritis studies [15, 17]. Persistent haematuria was defined as the presence of ≥ 5 RBC/HPF in at least three consecutive urine specimens obtained within the first 3 months after kidney biopsy. This definition was adapted from IgA nephropathy studies demonstrating the prognostic relevance of sustained haematuria rather than single‐timepoint assessment [15], and was chosen to minimise misclassification due to transient or biopsy‐related haematuria.

### 2.4 Histopathological Assessment

All kidney biopsy specimens were evaluated by experienced renal pathologists blinded to clinical outcomes. The histopathological chronicity score was calculated according to the standardised grading system proposed by Sethi et al. [18]. This system scores glomerulosclerosis (global and segmental) from 0 to 3 (< 10%, 10%–25%, 26%–50%, > 50%), tubular atrophy from 0 to 3, interstitial fibrosis from 0 to 3 and arteriosclerosis from 0 to 1. The total renal chronicity score (range: 0–10) grades overall severity into minimal (0–1), mild (2–4), moderate (5–7) and severe (≥ 8).

### 2.5 Outcomes

The primary outcome was ESKD, defined as initiation of chronic renal replacement therapy (haemodialysis, peritoneal dialysis or kidney transplantation). Patients were followed from the date of kidney biopsy until ESKD, death, loss to follow‐up or study end (December 2023).

### 2.6 Statistical Analyses

Descriptive statistics (number and percent, median and interquartile range [IQR]) were used to characterise participants with and without persistent haematuria. Comparison between groups was performed using the Mann–Whitney *U* test for continuous measures and chi‐squared or Fisher's exact test for categorical measures. Kidney survival was estimated using the Kaplan–Meier method with 95% CI calculated by the Greenwood formula. Survival curves were compared using the log‐rank test. Cox proportional hazards regression was performed to identify independent predictors of ESKD, with FSGS as the reference category for diagnosis given its known poor prognosis. Variables with *p* < 0.05 in univariate analysis were entered into the multivariate model. Because purely data‐driven selection can omit clinically important confounders, the model was additionally confirmed in a sensitivity analysis that forced a prespecified set of clinically and biologically relevant covariates (diagnosis, baseline eGFR, proteinuria and persistent haematuria) irrespective of univariate significance (Table S3); this yielded materially unchanged estimates. The proportional hazards assumption was verified for all covariates using scaled Schoenfeld residuals, with no violation detected (all *p* > 0.05). To account for the competing risk of death, a sensitivity analysis based on cumulative incidence functions (Grey test) and Fine‐Grey sub‐distribution hazard models was performed (Table S2 and Figure S2). Effect modification was assessed by fitting Cox models within prespecified subgroups (diagnosis, baseline eGFR, proteinuria, hypertension, age and sex) with formal haematuria‐by‐subgroup interaction terms, summarised as a forest plot (Figure S1 and Table S1). No patient reached ESKD, died or was lost to follow‐up during the 3‐month exposure‐assessment window, mitigating immortal time bias. Full results of the competing‐risk and subgroup analyses are reported in the Supporting Information (Tables S1–S3 and Figures S1–S2). A *p* value of < 0.05 was the criterion for statistical significance. Analyses were performed using R version 4.2.0.

## 3 Results

### 3.1 Baseline Characteristics of the Study Population

Participants with MN, MCD and FSGS (*N* = 236) were included in the study. Among all eligible participants, 114 (48%) had MN, 76 (32%) had MCD and 46 (20%) had FSGS. The median age was 50 (38–61) years and 141 (60%) were male. At baseline, 73 (31%) had persistent haematuria and 163 (69%) were negative for persistent haematuria. Those who had haematuria compared with those without haematuria were more likely to have hypertension (55% vs. 36%, *p* = 0.007) and had higher proteinuria (5.3 vs. 3.2 g/g, *p* = 0.01). There were no significant differences in age, sex, eGFR, diagnosis distribution or histological chronicity score between groups (Table 1).

**TABLE 1:** Baseline characteristics at kidney biopsy.

Variable	Total *N* = 236	Hematuria absent *n* = 163	Hematuria present *n* = 73	*p*
Age (year)	50 (38–61)	48 (37–59)	53 (40–63)	0.08
Men (%)	60	60	60	0.9
Primary diagnosis, *n* (%)				0.5
Membranous nephropathy	114 (48)	76 (47)	38 (52)	
Minimal change disease	76 (32)	56 (34)	20 (27)	
FSGS	46 (20)	31 (19)	15 (21)	
Charlson comorbidity score	2 (2–3)	2 (1–2)	2 (2–3)	0.06
Arterial hypertension, %	42	36	55	**0.007**
eGFR, mL/min/1.73 m^2^	65.5 (46–86)	68.8 (48–88)	62.1 (42–77)	0.08
Serum creatinine, mg/dL	1.1 (0.9–1.5)	1.1 (0.9–1.5)	1.2 (1.0–1.6)	0.09
Proteinuria (g/g)	3.9 (1.8–6.7)	3.2 (1.6–6.1)	5.3 (2.4–7.0)	**0.01**
Haemoglobin (g/dL)	13.6 (12.0–14.9)	13.8 (12.2–15.1)	13.1 (11.9–14.5)	0.1
Serum albumin (g/dL)	3.3 (2.8–4.0)	3.4 (2.7–4.0)	3.3 (2.8–4.0)	0.6
C‐reactive protein (mg/L)	3 (1–6)	3 (1–6)	3 (1–7)	0.7
Serum cholesterol (mg/dL)	295 (226–379)	295 (228–376)	296 (226–389)	0.8
Serum triglycerides (mg/dL)	199 (140–288)	190 (139–267)	223 (143–339)	0.3
Total renal chronicity score	0 (0–1)	0 (0–1)	0 (0–2)	0.6
Medication (%)				
ACEI/ARB	53	50	58	0.4
Immunosuppression	76	74	81	0.4
ESKD during follow‐up (%)	16	9	32	**< 0.001**
Death during follow‐up (%)	16	15	18	0.5

*Note:* Values are *n* (%) or median (interquartile range). The bold values means statisc significance ‐ value *p* < 0.05.

Abbreviations: ACEI/ARB, angiotensin‐converting enzyme inhibitor/angiotensin receptor blocker; eGFR, estimated glomerular filtration rate; ESKD, end‐stage kidney disease; FSGS, focal segmental glomerulosclerosis.

### 3.2 Incidence of Kidney Failure

The median follow‐up from baseline was 96 months (IQR: 75–113), with a maximum of 142 months. During this period, 38 patients (16%) reached ESKD. ESKD incidence varied significantly by diagnosis: FSGS 41% (19/46), MN 11% (12/114) and MCD 9% (7/76). ESKD incidence was significantly higher in patients with persistent haematuria compared with those without: 32% (23/73) versus 9% (15/163), *p* < 0.001. This association was consistent across all three podocytopathies: in MN, 24% (9/38) versus 4% (3/76); in MCD, 25% (5/20) versus 4% (2/56); and in FSGS, 60% (9/15) versus 32% (10/31).

### 3.3 Kaplan–Meier Survival Analysis

**FIGURE 1:**
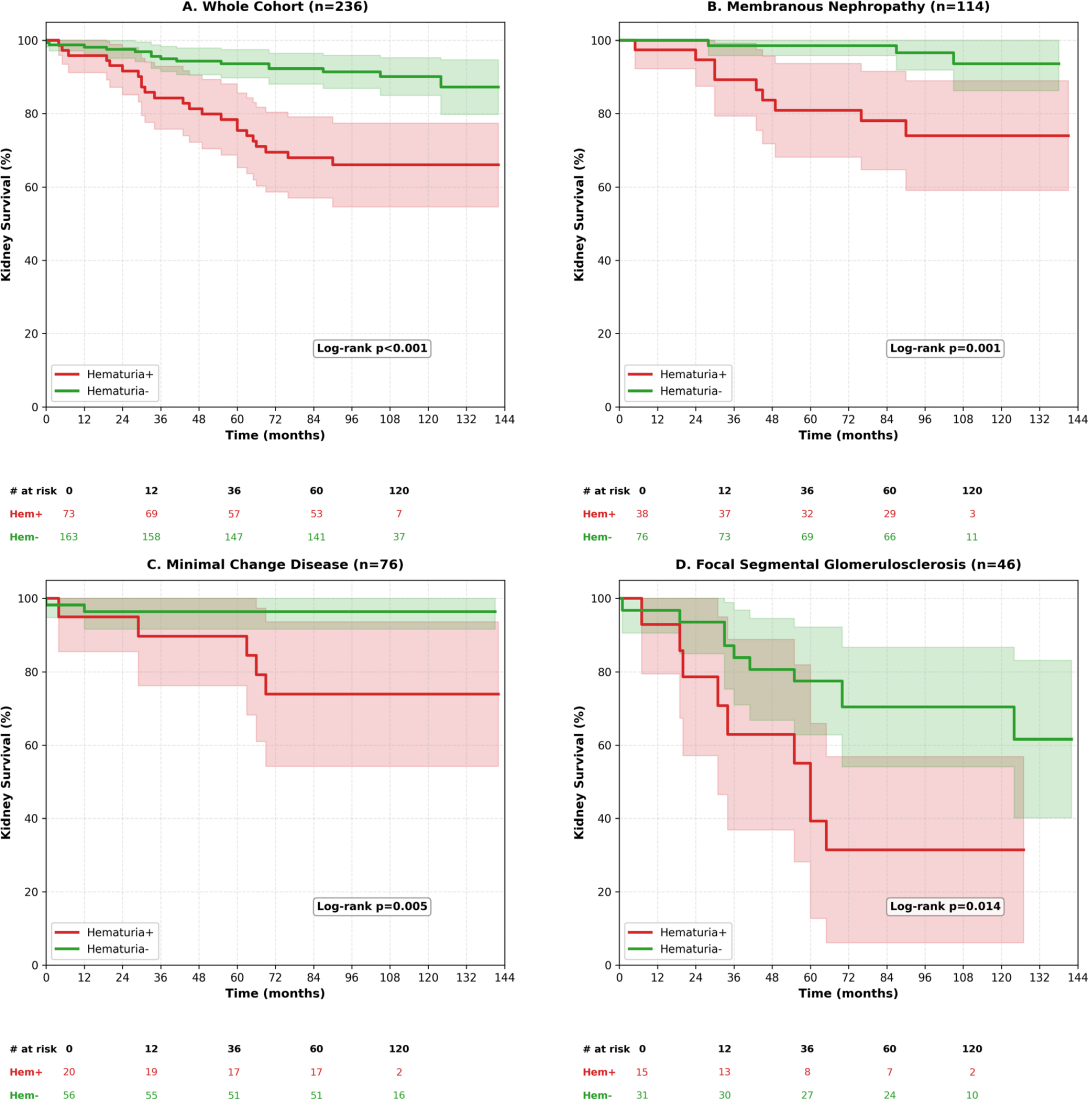
Kaplan–Meier kidney survival estimates by persistent hematuria status. Survival estimates for the primary outcome (end‐stage kidney disease requiring renal replacement therapy) are shown for patients with persistent hematuria (red lines) versus those without persistent hematuria (green lines). Shaded areas represent 95% confidence intervals calculated using the Greenwood formula. (A) Whole cohort (*N* = 236). Five‐year kidney survival was 75.4% (95% CI: 65.3–85.6) in the hematuria group versus 93.6% (95% CI: 89.8–97.4) in the no‐hematuria group; 10‐year kidney survival was 66.0% (95% CI 54.6–77.4) versus 90.1% (95% CI: 85.0–95.2). Log‐rank *p* < 0.001. (B) Membranous nephropathy (*n* = 114). Log‐rank *p* = 0.001. (C) Minimal change disease (*n* = 76). Log‐rank *p* = 0.005. (D) Focal segmental glomerulosclerosis (*n* = 46). Log‐rank *p* = 0.014. Numbers at risk are shown below each panel at 0, 12, 36, 60 and 120 months of follow‐up.

Unadjusted Kaplan–Meier curves demonstrate that the presence of persistent haematuria was associated with significantly worse kidney survival (log‐rank *p* < 0.001) (Figure 1A). Five‐year kidney survival rates were 75.4% (95% CI: 65.3–85.6) versus 93.6% (95% CI: 89.8–97.4). At 10 years, kidney survival rates were 66.0% (95% CI: 54.6–77.4) versus 90.1% (95% CI: 85.0–95.2). The associations were similar when stratified by diagnosis (Figure 1B–D).

### 3.4 Cox Proportional Hazards Regression

In univariate analysis, persistent haematuria was strongly associated with ESKD (HR: 4.02; 95% CI: 2.09–7.73; *p* < 0.001). Other significant predictors included lower eGFR, higher proteinuria, higher chronicity score and higher Charlson comorbidity score. Age, ACEI/ARB use and immunosuppression were not significantly associated with ESKD in univariate analysis. With FSGS as the reference category, both MN (HR: 0.48; 95% CI: 0.24–0.96; *p* = 0.04) and MCD (HR: 0.44; 95% CI: 0.19–0.99; *p* = 0.05) were associated with better kidney survival. In multivariate analysis adjusting for diagnosis, eGFR, proteinuria, Charlson score and chronicity score, persistent haematuria remained independently associated with ESKD (HR: 3.55; 95% CI: 1.78–7.05; *p* < 0.001). MN and MCD remained associated with significantly lower risk of ESKD compared with FSGS (Table 2). The proportional hazards assumption was satisfied for all covariates (all Schoenfeld *p* > 0.05; Supporting Information). The independent association of persistent haematuria with ESKD was preserved in the prespecified‐covariate sensitivity model (HR: 3.63; 95% CI: 1.83–7.22; Table S3) and in the competing‐risk analysis accounting for the competing risk of death (cause‐specific HR: 3.84, 95% CI: 1.98–7.44; Fine–Grey sub‐distribution HR: 3.60, 95% CI: 1.91–6.77; both *p* < 0.001; Table S2 and Figure S2). The cumulative incidence of ESKD at 120 months was 31.9% in patients with persistent haematuria versus 9.3% in those without (Grey test *p* < 0.001). The association was directionally consistent across all examined subgroups, with no significant haematuria‐by‐subgroup interaction (all interaction *p* ≥ 0.08; Figure S1 and Table S1).

**TABLE 2:** Cox proportional hazards regression for ESKD.

Variable	Univariate HR (95% CI)	*p*	Multivariate HR (95% CI)	*p*
Persistent hematuria (yes vs. no)	4.02 (2.09–7.73)	**< 0.001**	3.55 (1.78–7.05)	**< 0.001**
Age (per 1 year)	1.01 (0.98–1.03)	0.61	—	—
eGFR (per 10 mL/min/1.73 m^2^)	0.64 (0.55–0.74)	**< 0.001**	0.70 (0.58–0.84)	**< 0.001**
Proteinuria (per 1 g/g)	1.08 (1.00–1.16)	**0.04**	1.11 (1.01–1.23)	**0.03**
MN vs. FSGS (reference)	0.48 (0.24–0.96)	**0.04**	0.24 (0.09–0.65)	**0.005**
MCD vs. FSGS (reference)	0.44 (0.19–0.99)	**0.05**	0.17 (0.06–0.52)	**0.002**
Chronicity score (per 1 point)	1.28 (1.17–1.39)	**< 0.001**	1.01 (0.89–1.15)	0.87
Charlson score (per 1 point)	1.27 (1.07–1.52)	**0.008**	1.12 (0.88–1.42)	0.36
ACEI/ARB (yes vs. no)	0.81 (0.43–1.52)	0.51	—	—
Immunosuppression (yes vs. no)	0.89 (0.43–1.83)	0.75	—	—

*Note:* Variables with *p* < 0.05 in univariate analysis were included in the multivariate model. The bold values means statisc significance ‐value *p* < 0.05.

Abbreviations: ACEI/ARB, angiotensin‐converting enzyme inhibitor/angiotensin receptor blocker; CI, confidence interval; eGFR, estimated glomerular filtration rate; ESKD, end‐stage kidney disease; FSGS, focal segmental glomerulosclerosis; HR, hazard ratio; MCD, minimal change disease; MN, membranous nephropathy.

## 4 Discussion

In a large cohort of participants with podocytopathies, namely MN, MCD and FSGS, we demonstrated that the presence of persistent haematuria early in the disease course was independently associated with a higher risk of ESKD and a 3.5‐fold increased risk after adjustment for traditional prognostic factors including eGFR, proteinuria, diagnosis, Charlson comorbidity score and histological chronicity. Because haematuria, unlike proteinuria, is usually not a factor in clinical management decision making, the presence of this urinary abnormality may be a valuable marker for kidney survival in podocytopathies.

Previous reports document haematuria as a common finding in proteinuric kidney disease [6]. At diagnosis, among 352 children with MCD and 31 children with FSGS, 22.7% and 48.4%, respectively, had haematuria [13]. A recent study of 287 children with new‐onset idiopathic nephrotic syndrome reported 42.5% demonstrating microscopic haematuria [11]. Fewer studies in adults reported the prevalence of haematuria‐by‐disease. In 410 adult patients in India, haematuria was present in 20.8% (21/101) with FSGS, 11.2% (11/98) with MCD and 25% (23/92) with MN [12]. Among 95 adults with MCD, 28.9% had haematuria at presentation [14]. The reported prevalence in our study (31%) is consistent with these reports, with a lower prevalence in MCD compared with FSGS or MN. In our cohort, it was more common in those with higher levels of proteinuria and in patients with hypertension.

Haematuria has been shown to be associated with kidney survival outcomes in a variety of large screening programmes. In a study of 1.2 million young Israeli adults without known kidney disease, the presence of persistent, asymptomatic, microscopic haematuria was significantly associated with the incidence of kidney failure after multivariable adjustments (HR: 18.5; 95% CI: 12.4–27.6) [4]. In a Japanese population‐based screening programme, adults with haematuria by dipstick testing had significantly higher odds of reaching kidney failure after adjustments [3]. We extend these findings to podocytopathies where persistent haematuria early in the disease course is significantly and independently associated with ESKD after adjusting for diagnosis, disease activity, eGFR and proteinuria.

A recent analysis from the NEPTUNE and CureGN cohorts by Marchel et al. [19] examined haematuria in 1516 participants with primary podocytopathies and found that haematuria at a single timepoint was associated with adverse outcomes (HR: 1.31 for the composite endpoint of kidney failure or 40% eGFR decline) and reduced likelihood of proteinuria remission (HR: 0.80). However, that study used single‐timepoint urinalysis assessment with a median time from biopsy to first urinalysis of 260 days. Our study's use of persistent haematuria, defined by ≥ 3 consecutive positive specimens within 3 months of biopsy, likely captured a more clinically relevant phenotype of sustained glomerular injury, resulting in a substantially stronger association with ESKD (HR: 3.55). This methodological difference highlights the importance of defining haematuria appropriately in clinical studies and suggests that isolated single‐timepoint assessment may underestimate the true prognostic impact.

In MN, haematuria has traditionally been considered uncommon, and the disease typically presents with nephrotic syndrome and a ‘bland’ urinary sediment. However, emerging evidence challenges this paradigm. Chen et al. [20] demonstrated that in patients with primary MN and nephrotic syndrome, higher levels of initial haematuria were associated with a 1.43‐fold greater hazard of relapse after adjusting for confounders. Furthermore, time‐averaged haematuria and cumulative duration of haematuria were also significantly associated with both relapse and renal progression. Nie et al. [21] found that in MN patients with sub‐nephrotic proteinuria, initial microhaematuria was an independent predictor of progression to nephrotic‐range proteinuria (HR: 1.11 per RBC/HPF, *p* < 0.001). Our finding that haematuria in MN carries a substantially increased risk of ESKD (24% vs. 4%) aligns with these observations and suggests that the traditional view of MN as a disease with bland sediment may need reconsideration.

While MCD is classically described as presenting without haematuria, microscopic haematuria is actually detected in 10%–30% of cases, and up to 58% of adult MCD patients in some series [14, 22]. Lionaki et al. [22] reported that 58.5% of 106 adults with MCD had microscopic haematuria at presentation. Importantly, MCD with haematuria may represent a distinct clinical phenotype. The presence of haematuria, hypertension and renal insufficiency in adults with MCD can make it clinically indistinguishable from FSGS, and some authors have suggested that MCD with persistent haematuria may actually represent early or sampling‐missed FSGS.

Our finding of markedly increased ESKD risk in MCD patients with haematuria (25% vs. 4%) is particularly striking given that MCD is traditionally considered to have an excellent prognosis with < 5% progressing to ESKD [23]. Indeed, large adult MCD series report progression to ESKD in fewer than 5% of patients, with such progression frequently attributed to an alternative or unsampled diagnosis of FSGS rather than to MCD itself. Against this background, the 25% ESKD rate we observed in MCD patients with persistent haematuria is markedly higher than expected and should be interpreted with caution: it may reflect either a genuinely high‐risk hematuric phenotype or, alternatively, the inclusion of unsampled FSGS within the haematuria‐positive MCD group. Although this association was strong, the small number of events (5 ESKD in 20 MCD patients with haematuria) warrants cautious interpretation and validation in larger cohorts.

FSGS has been recognised to present with microscopic haematuria in 30%–50% of patients. Korbet [24] reported that 32.6% of adults with primary FSGS presented with microscopic haematuria. The presence of haematuria in FSGS may reflect more severe glomerular injury involving not just podocyte damage but also glomerular capillary wall disruption. The collapsing variant of FSGS, which is associated with the worst prognosis, often presents with haematuria alongside severe proteinuria and rapid progression to ESKD [25]. Our finding that 60% of FSGS patients with haematuria progressed to ESKD compared with 32% without haematuria underscores the clinical significance of this sign, although the smaller sample size (*n* = 46) limits statistical precision.

The underlying mechanism for these associations is uncertain but likely involves disruption of the intricate crosstalk between podocytes and glomerular endothelial cells. Glomerular haematuria suggests a defect in the glomerular basement membrane (GBM) that red blood cells traverse during passage through the glomerular capillary and into the urinary space. Therefore, the extent of haematuria in patients with podocytopathies may indicate the severity of filtration barrier damage. In our cohort, erythrocytes were predominantly dysmorphic in patients with haematuria, supporting the glomerular origin of the red blood cells. Vascular endothelial growth factor A (VEGF‐A) secreted by podocytes plays a central role in maintaining endothelial cell integrity. Studies by Veron et al. [26] demonstrated that podocyte‐specific VEGF‐A knockdown results in endothelial swelling, GBM lamination, mesangiolysis and rapid proteinuria—phenotypes resembling thrombotic microangiopathy. Hartleben et al. [27] showed that BECLIN1 is essential for podocyte secretory function and VEGF‐A release, and that podocyte‐specific Beclin1 deletion leads to early‐onset glomerulosclerosis through impaired endothelial support. In podocytopathies, injured podocytes may exhibit dysregulated VEGF‐A expression, disrupting endothelial homeostasis. When podocyte‐endothelial crosstalk is compromised, endothelial cells lose their protective glycocalyx, become more permeable and may undergo apoptosis [28]. The resulting glomerular capillary wall defects could explain the presence of haematuria. Additional mechanisms include tubular heme toxicity, wherein filtered haemoglobin and heme proteins induce oxidative stress, inflammation and tubulointerstitial fibrosis [9, 10, 29]. The finding of higher baseline proteinuria in patients with haematuria supports the concept that these patients have more severe glomerular injury involving both the epithelial and endothelial layers.

One intriguing question is whether an underlying genetic polymorphism, such as genes associated with GBM composition and structure, may explain the presence of haematuria in some patients and confer a higher risk of disease progression. In a study of over 3000 patients with mixed aetiologies of CKD, including glomerular disease, 66 monogenic disorders were detected in 307 patients, of which 30% were collagen IV gene variants [30]. In a smaller cohort of patients with biopsy‐proven FSGS who had undergone whole‐exome sequencing, pathogenic variants in COL4A (A3/4/5) accounted for 55% of patients attributed to a single‐gene cause [31]. A genetic predisposition for GBM structural abnormalities, therefore, may contribute to kidney disease progression in those without a clinical diagnosis of Alport syndrome or thin basement membrane disease.

Strengths of this study include the large number of participants with comprehensive clinical information and laboratory tests and sufficient follow‐up with clinical events to enable a meaningful assessment of the effect of haematuria. The long median follow‐up of 96 months (8 years) with a maximum of 12 years allowed adequate time to observe the development of ESKD, which is a hard, unequivocal endpoint. The definition of persistent haematuria using three consecutive positive specimens adds biological plausibility, as it excludes transient haematuria that may occur due to intercurrent illness, menstruation or biopsy‐related bleeding. All diagnoses were biopsy‐proven with centralised histopathological review, ensuring diagnostic accuracy. The chronicity score was assessed using the standardised grading system proposed by Sethi et al. [18]. The consistent direction of effect across all three podocytopathies strengthens generalisability.

However, there are important limitations. The retrospective single‐centre design may limit generalisability and selection bias cannot be excluded. We did not assess time‐varying haematuria or haematuria remission during follow‐up, which would provide additional insights into whether resolution of haematuria is associated with improved outcomes, as has been demonstrated in IgA nephropathy [15]. Proteinuria remission rates were not evaluated—an important limitation as immunological remission strongly influences prognosis in podocytopathies [32]. The multivariable model adjusted for first‐line immunosuppressive therapy at baseline but did not incorporate treatment response, remission, relapse or longitudinal changes in proteinuria over follow‐up. Persistent haematuria may therefore partly reflect ongoing disease activity rather than acting as a fully independent prognostic factor and we acknowledge this explicitly. Furthermore, although persistent haematuria was defined using three consecutive specimens within the first 3 months after biopsy and analysed as a fixed baseline exposure, haematuria is a dynamic phenomenon that may remit or recur; this could introduce exposure misclassification over the long follow‐up. No patient reached ESKD, died or was lost to follow‐up before completion of the three‐month exposure‐assessment window, which mitigates immortal time bias; however, uniform longitudinal urinalysis data beyond this window were not available, precluding time‐varying or time‐averaged haematuria models analogous to those reported in IgA nephropathy. The FSGS subgroup was relatively small (*n* = 46), resulting in wide confidence intervals for some estimates. We did not have data on novel biomarkers such as anti‐PLA2R antibodies for MN [33] or genetic testing. There was no information available on other conditions that cause haematuria, such as kidney/bladder malignancies or nephrolithiasis. However, known malignancy is an exclusion criterion and prevalence was similar in male and female patients. Finally, the mean duration of follow‐up is substantial; however, more extended observation is warranted to substantiate our findings and to assess the relevance of serial assessments of haematuria over the course of follow‐up. We deliberately used ESKD requiring chronic kidney replacement therapy as the primary outcome because it is a hard, unequivocal and uniformly ascertained endpoint. Intermediate kidney‐function endpoints such as annual eGFR slope or a sustained 40%–50% eGFR decline would require densely sampled longitudinal creatinine measurements, which were not available in a uniform manner across this retrospective cohort; ESKD over a median follow‐up of 8 years therefore represented the most robust and least bias‐prone outcome. While ESKD is comparatively uncommon in MN and MCD, its consistent association with persistent haematuria across all three diagnoses argues against the result being driven solely by FSGS.

## 5 Conclusions

Persistent haematuria is relatively common in patients with podocytopathies and is associated with a higher risk of ESKD. Urinalysis is widely available, is inexpensive and can be easily incorporated into clinical practice, providing a valuable, easily accessible data point to help in formulating patient prognosis. Future work is warranted to determine whether qualitative changes in haematuria reflect parallel changes in the likelihood of disease progression and to discover the underlying mechanisms to guide development of therapeutic interventions.

## Author Contributions

**Gabriel Ștefan:** conception and design, acquisition and interpretation of data, drafting the article. **Nicoleta Petre:** acquisition of data, assisted in drafting specific sections of the manuscript, critical revision of the article. **Adrian Zugravu:** acquisition and interpretation of data, critical revision of the article. **Simona Stancu:** conception and design, acquisition and interpretation of data, coordinated the critical revisions and integrated feedback from all authors. All authors have read and approved the final version of the manuscript and agree to be accountable for the integrity and accuracy of all aspects of the work.

## Funding

The authors have nothing to report.

## Ethics Statement

This study was conducted in accordance with the 1964 Declaration of Helsinki and its later amendments and was approved by the Ethics Committee of Dr. Carol Davila Teaching Hospital of Nephrology (No 34/2025).

## Conflicts of Interest

The authors declare no conflicts of interest.

## Supporting information


**Data S1:** Supplementary methods.
**Table S1:** Subgroup analysis of persistent haematuria and ESKD with interaction tests.
**Table S2:** Competing‐risk analysis (cause‐specific and Fine–Grey models).
**Table S3:** Covariate‐selection sensitivity analysis (prespecified‐covariate model).
**Figure S1:** Forest plot of subgroup hazard ratios.
**Figure S1:** Hazard ratios (squares) and 95% confidence intervals (horizontal lines) for the association between the exposure and ESKD within prespecified subgroups, with HR (95% CI) labelled at right.
**Figure S2:** Cumulative incidence of ESKD with death as a competing event.
**Figure S2:** Cumulative incidence function of ESKD, treating death as a competing event, in patients with (red) and without (blue) the exposure, estimated by the Aalen–Johansen method. Separation of the curves indicates a higher absolute incidence of ESKD in the exposed group that is not attributable to differential mortality.

## Data Availability

The data that support the findings of this study are available from the corresponding author upon reasonable request.
