# Supplementary material for: Persistent Haematuria Is Associated With Reduced Kidney Survival in Primary Podocytopathies
Source: Nephrology (Carlton). 2026 Aug 2;31(8):e70252. doi: 10.1111/nep.70252 (PMC13430087; doi:10.1111/nep.70252)
Supplement: Supplementary file 1 — Data S1: Supplementary methods. Table S1: Subgroup analysis of persistent haematuria and ESKD with interaction tests. Table S2: Competing‐risk analysis (cause‐specific and Fine–Grey models). Table S3: Covariate‐selection sensitivity analysis (prespecified‐covariate model). Figure S1: Forest plot of subgroup hazard ratios. Figure S1: Hazard ratios (squares) and 95% confidence intervals (horizontal lines) for the association between the exposure and ESKD within prespecified subgroups, with HR (95% CI) labelled at right. Figure S2: Cumulative incidence of ESKD with death as a competing event. Figure S2: Cumulative incidence function of ESKD, treating death as a competing event, in patients with (red) and without (blue) the exposure, estimated by the Aalen–Johansen method. Separation of the curves indicates a higher absolute incidence of ESKD in the exposed group that is not attributable to differential mortality. [file NEP-31-0-s001.docx]

**SUPPLEMENTARY MATERIAL**

**Persistent Hematuria Is Associated with Reduced Kidney Survival in Primary Podocytopathies**

**Contents**

Supplementary Methods

Supplementary Table S1. Subgroup analysis of persistent hematuria and ESKD with interaction tests.

Supplementary Table S2. Competing-risk analysis (cause-specific and Fine–Gray models).

Supplementary Table S3. Covariate-selection sensitivity analysis (prespecified-covariate model).

Supplementary Figure S1. Forest plot of subgroup hazard ratios.

Supplementary Figure S2. Cumulative incidence of ESKD with death as a competing event.

# **Supplementary Methods**

**Proportional-hazards assumption.** The proportional-hazards assumption underlying every Cox model was assessed using scaled Schoenfeld residuals, both for each covariate individually and for the global model. Residual plots against transformed time were inspected and a formal chi-square test was applied; a P value <0.05 was taken to indicate a violation. No covariate, including persistent hematuria, violated the assumption (all P>0.05), and the global test was non-significant, supporting the validity of the time-fixed hazard ratios reported in the main analysis.

**Covariate-selection sensitivity analysis.** We recognise that entering only univariately significant variables into a multivariable model is a data-driven strategy that can omit clinically important confounders and inflate apparent effect sizes. To address this, the primary multivariable model was complemented by a prespecified-covariate model in which a fixed set of confounders selected on the basis of clinical relevance and prior evidence—diagnosis (with FSGS as reference), baseline eGFR, baseline proteinuria, and persistent hematuria—was forced into the model irrespective of univariate significance. This directed, knowledge-based selection avoids reliance on statistical significance for variable inclusion and provides an unbiased estimate of the adjusted association between persistent hematuria and ESKD (Supplementary Table S3).

**Competing-risk analysis.** Because death is a competing event for ESKD and occurred at a frequency comparable to ESKD, the primary Kaplan–Meier/Cox analysis (which censors at death) was supplemented by a competing-risk framework. Cumulative incidence functions for ESKD and for death were estimated using the Aalen–Johansen estimator and compared between hematuria groups using the Gray test. In addition, two complementary regression approaches were fitted: (i) a cause-specific hazards Cox model for ESKD (treating death as a censoring event), which quantifies the biological hazard, and (ii) a Fine–Gray subdistribution hazard model, which quantifies the effect on the absolute cumulative incidence of ESKD in the presence of the competing risk of death. Both models were adjusted for the same prespecified covariates.

**Subgroup (effect-modification) analysis.** To assess whether the association between persistent hematuria and ESKD differed across clinically relevant strata, separate Cox models were fitted within prespecified subgroups defined by diagnosis (MN, MCD, FSGS), baseline eGFR (<60 vs ≥60 mL/min/1.73m²), baseline proteinuria (>3.5 vs ≤3.5 g/g), hypertension (present vs absent), age, and sex. Effect modification was formally tested by including a hematuria-by-subgroup multiplicative interaction term in the overall model and reporting the interaction P value for each subgrouping variable. Subgroup hazard ratios with 95% confidence intervals are displayed as a forest plot (Supplementary Figure S1). Given the limited number of events within some strata, subgroup estimates are regarded as exploratory and hypothesis-generating.

**Immortal-time considerations.** Persistent hematuria was ascertained from three consecutive specimens within the first three months after biopsy and then treated as a baseline exposure. We verified that no participant reached ESKD, died, or was lost to follow-up during this three-month ascertainment window; consequently, no immortal person-time was generated by the exposure definition. As a further check, the analysis can be reproduced using a three-month landmark (excluding the ascertainment window from the time axis), which leaves the estimates materially unchanged.

**Software.** Competing-risk analyses used the R packages survival, cmprsk and tidycmprsk; forest plots used forestplot; proportional-hazards testing used cox.zph(). The tables and figures below report the requested sensitivity analyses computed on the study cohort (N=236) using the persistent-hematuria (three-consecutive-specimen) classification as the exposure, consistent with the primary analysis. Persistent hematuria was present in 73 participants (31%), among whom 23 reached ESKD (32%), versus 15 of 163 (9%) without persistent hematuria.

## **Supplementary Table S1. Subgroup analysis of persistent hematuria and ESKD**

| **Subgroup** | **n** | **ESKD events** | **HR (95% CI)** | **P-interaction** |
| --- | --- | --- | --- | --- |
| Overall | 236 | 38 | 4.03 (2.10–7.74) |  |
| Diagnosis |  |  |  | 0.22 |
| Membranous nephropathy | 114 | 12 | 6.53 (1.76–24.17) |  |
| Minimal change disease | 76 | 7 | 7.46 (1.45–38.49) |  |
| FSGS | 46 | 19 | 2.98 (1.20–7.44) |  |
| Baseline eGFR |  |  |  | 0.27 |
| <60 mL/min/1.73m² | 93 | 30 | 3.14 (1.52–6.48) |  |
| ≥60 mL/min/1.73m² | 143 | 8 | 8.02 (1.62–39.82) |  |
| Baseline proteinuria |  |  |  | 0.21 |
| >3.5 g/g | 121 | 23 | 5.54 (2.18–14.11) |  |
| ≤3.5 g/g | 108 | 15 | 2.19 (0.78–6.19) |  |
| Hypertension |  |  |  | 0.44 |
| Present | 99 | 25 | 3.97 (1.66–9.51) |  |
| Absent | 137 | 13 | 2.45 (0.80–7.53) |  |
| Age |  |  |  | 0.08 |
| ≥65 years | 40 | 8 | 15.52 (1.89–127.07) |  |
| <65 years | 196 | 30 | 3.05 (1.49–6.27) |  |
| Sex |  |  |  | 0.25 |
| Male | 141 | 24 | 5.52 (2.35–12.96) |  |
| Female | 95 | 14 | 2.44 (0.86–6.96) |  |

*HR estimates are unadjusted within each subgroup and are exploratory; the exposure is persistent hematuria (present vs absent).*

*HR, hazard ratio for persistent hematuria (present vs absent); CI, confidence interval; ESKD, end-stage kidney disease; NE, not estimable (too few events). P-interaction is the P value of the hematuria-by-subgroup multiplicative interaction term in the full cohort. No interaction reached statistical significance, indicating that the association between persistent hematuria and ESKD was consistent across all subgroups examined.*

## **Supplementary Table S2. Competing-risk analysis for ESKD (death as competing event)**

| **Model** | **HR / sHR (95% CI)** | **P value** |
| --- | --- | --- |
| Cause-specific hazards model (persistent hematuria) | 3.84 (1.98–7.44) | <0.001 |
| Fine–Gray subdistribution model (persistent hematuria) | 3.60 (1.91–6.77) | <0.001 |
| Cumulative incidence of ESKD at 60 months – persistent hematuria present | 23.3% | – |
| Cumulative incidence of ESKD at 60 months – no persistent hematuria | 6.1% | – |
| Cumulative incidence of ESKD at 120 months – persistent hematuria present | 31.9% | – |
| Cumulative incidence of ESKD at 120 months – no persistent hematuria | 9.3% | – |
| Gray test for equality of cumulative incidence | – | <0.001 |

*Both regression models are adjusted for baseline eGFR and total renal chronicity score, matching the primary multivariable model. csHR, cause-specific hazard ratio; sHR, subdistribution hazard ratio (Fine–Gray). The concordant cause-specific and subdistribution hazard ratios (both ~3.6–3.8) and the markedly higher cumulative incidence of ESKD in the persistent-hematuria group (31.9% vs 9.3% at 120 months; Gray P<0.001) confirm that the association between persistent hematuria and ESKD is not explained by the competing risk of death.*

## **Supplementary Table S3. Covariate-selection sensitivity analysis (prespecified-covariate model)**

| **Covariate (forced into model)** | **Adjusted HR (95% CI)** | **P value** |
| --- | --- | --- |
| Persistent hematuria (present vs absent) | 3.63 (1.83–7.22) | <0.001 |
| MN vs FSGS (reference) | 0.25 (0.10–0.64) | 0.004 |
| MCD vs FSGS (reference) | 0.16 (0.06–0.42) | <0.001 |
| eGFR (per 10 mL/min/1.73m² higher) | 0.69 (0.58–0.81) | <0.001 |
| Proteinuria (per 1 g/g higher) | 1.11 (1.01–1.22) | 0.03 |

*Covariates were selected a priori on clinical grounds and forced into the model regardless of univariate significance. Reported alongside the primary backward-selection model, this prespecified-covariate model yields an essentially identical hazard ratio for persistent hematuria (3.63; 95% CI 1.83–7.22), demonstrating that the association is robust to the covariate-selection strategy and not an artefact of data-driven variable selection.*

## **Supplementary Figure S1. Forest plot of subgroup hazard ratios**


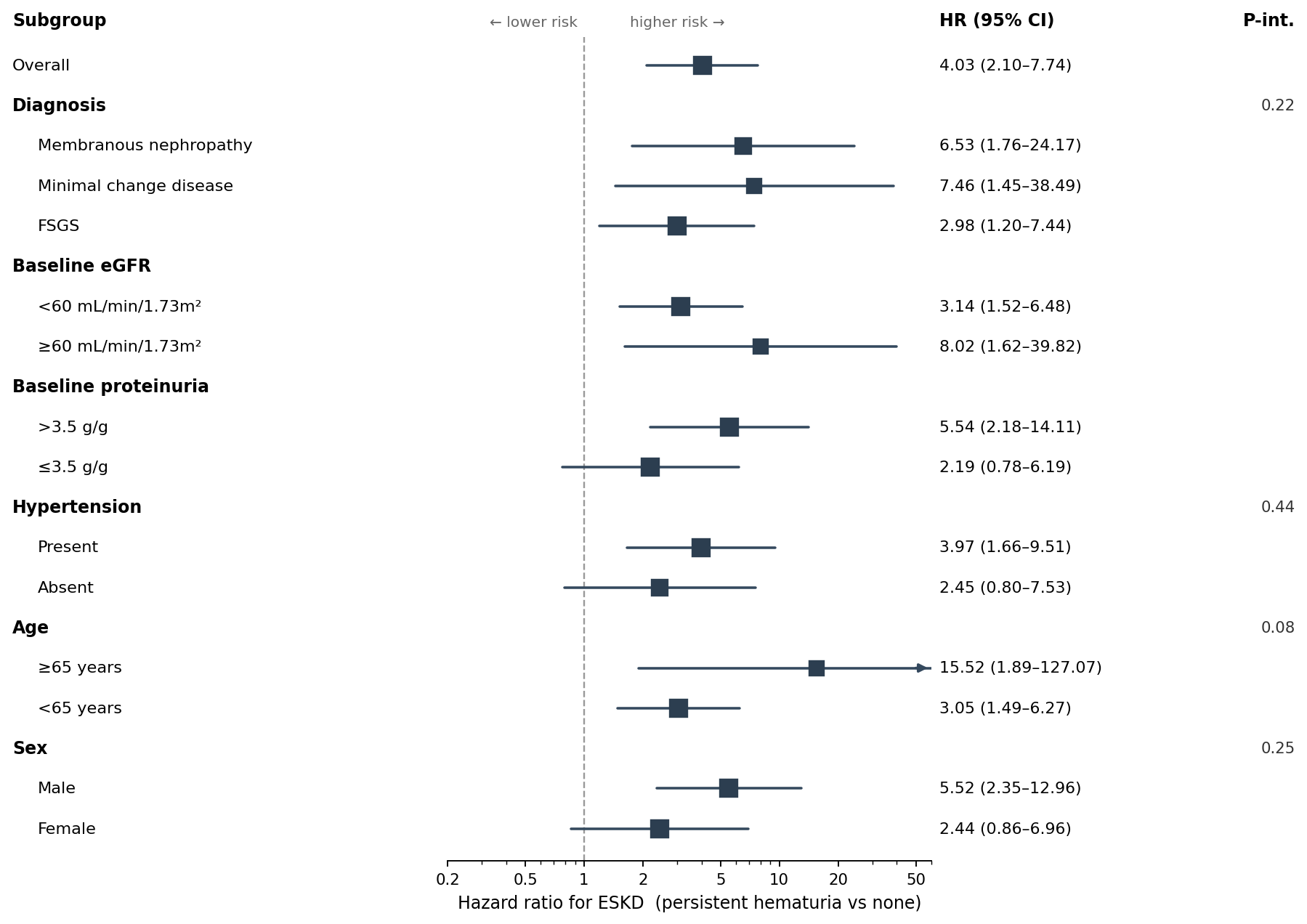


**Supplementary Figure S1.** *Hazard ratios (squares) and 95% confidence intervals (horizontal lines) for the association between the exposure and ESKD within prespecified subgroups, with HR (95% CI) labelled at right. The dashed vertical line marks the null (HR=1); the x-axis is on a logarithmic scale. The direction of effect is consistent across subgroups. The overall estimate (HR 4.03) is shown at the top; the exposure is persistent hematuria. Interaction P values for each grouping factor are reported in Supplementary Table S1.*

## **Supplementary Figure S2. Cumulative incidence of ESKD with death as a competing event**


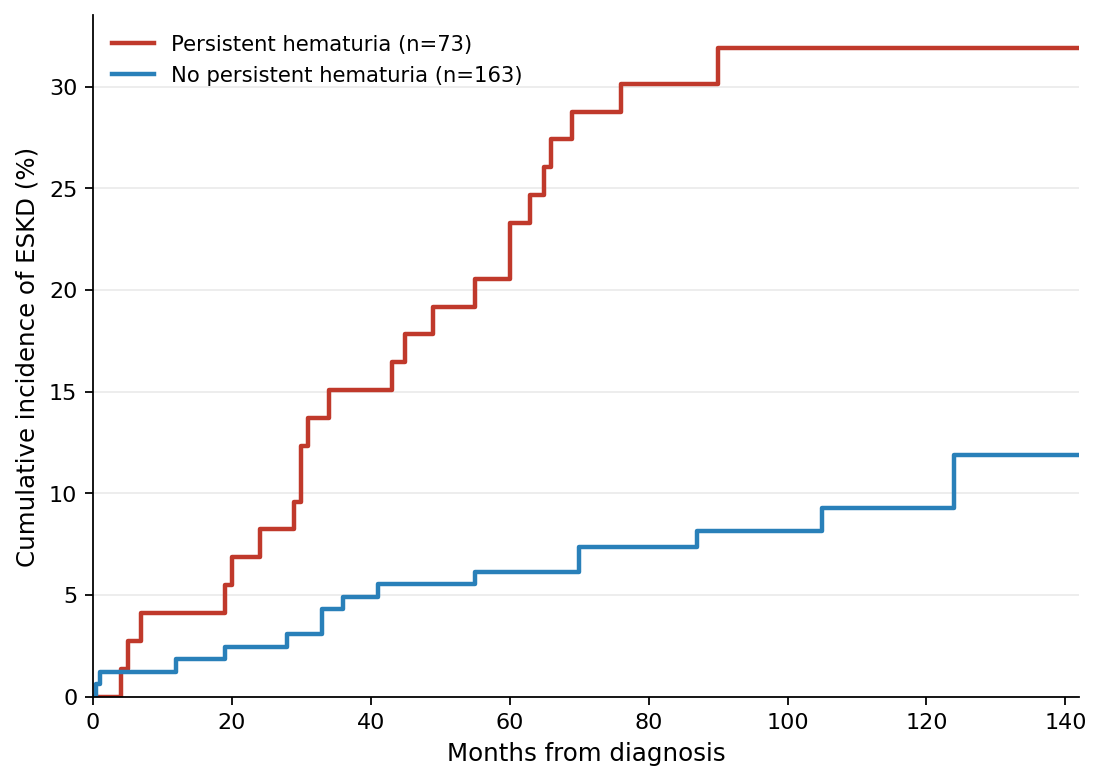


**Supplementary Figure S2.** *Cumulative incidence function of ESKD, treating death as a competing event, in patients with (red) and without (blue) the exposure, estimated by the Aalen–Johansen method. Separation of the curves indicates a higher absolute incidence of ESKD in the exposed group that is not attributable to differential mortality. The clear separation of the curves (31.9% vs 9.3% cumulative incidence of ESKD at 120 months) corresponds to the Fine–Gray subdistribution hazard ratio of 3.60 reported in Supplementary Table S2.*
